# Thymoma Associated Myasthenia Gravis (TAMG): Differential Expression of Functional Pathways in Relation to MG Status in Different Thymoma Histotypes

**DOI:** 10.3389/fimmu.2020.00664

**Published:** 2020-04-16

**Authors:** Yosuke Yamada, Cleo-Aron Weis, Julian Thelen, Carsten Sticht, Berthold Schalke, Philipp Ströbel, Alexander Marx

**Affiliations:** ^1^Institute of Pathology, University Medical Centre Mannheim, Heidelberg University, Mannheim, Germany; ^2^Medical Faculty Mannheim, Medical Research Center, Heidelberg University, Mannheim, Germany; ^3^Department of Neurology, University of Regensburg, Regensburg, Germany; ^4^Institute of Pathology, University Medical Center Göttingen, University of Göttingen, Göttingen, Germany

**Keywords:** thymoma, myasthenia gravis, autoimmunity, the cancer genome atlas (TCGA), functional pathways, metabolism, macrophage polarization

## Abstract

A unique feature of thymomas is their unrivaled frequency of associated myasthenia gravis (MG). Previous studies reported that MG+ thymomas contain a larger number of mature “pre-emigrant” CD4+ T cells than MG- thymomas and that most thymomas do not contain AIRE expressing cells irrespective of MG status. These findings suggest that CD4+ T cells that mature inside the abnormal microenvironment of thymomas and egress to the blood are critical to the development of thymoma-associated MG (TAMG) irrespective of thymoma histotype. However, underlying mechanisms have remained enigmatic. To get hints to mechanisms underlying TAMG, we pursue three hypotheses: (i) Functional pathways with metabolic and immunological relevance might be differentially expressed in TAMG(+) compared to TAMG(-) thymomas; (ii) differentially enriched pathways might be more evident in immature lymphocyte-poor (i.e., tumor cell/stroma-rich) thymoma subgroups; and (iii) mechanisms leading to TAMG might be different among thymoma histological subtypes. To test these hypotheses, we compared the expression of functional pathways with potential immunological relevance (*N* = 380) in relation to MG status separately in type AB and B2 thymomas and immature lymphocyte-rich and lymphocyte-poor subgroups of these thymoma types using the TCGA data set. We found that <10% of the investigated pathways were differentially upregulated or downregulated in MG+ compared to MG- thymomas with significant differences between AB and B2 thymomas. The differences were particularly evident, when epithelial cell/stroma-rich subsets of type AB and B2 thymomas were analyzed. Unexpectedly, some MG-associated pathways that were significantly upregulated in AB thymomas were significantly downregulated in B2 thymomas, as exemplified by the oxidative phosphorylation pathway. Conversely, the MG-associated pathway related to macrophage polarization was downregulated in MG+ AB thymoma and upregulated in MG+ B2 thymoma. We conclude that functional pathways are significantly associated with TAMG, and that some mechanisms leading to TAMG might be different among thymoma histological subtypes. Functions related to metabolisms, vascular and macrophage biology are promising new candidate mechanisms potentially involved in the pathogenesis of TAMG. More generally, the results imply that future studies addressing pathomechanisms of TAMG should take the histotype and abundance of tumor cells and non-neoplastic stromal components of thymomas into account.

## Introduction

Thymomas are tumors that appear to be derived from or show differentiation toward thymic epithelial cells, with a resemblance to the normal thymic histological architecture, such as discrete lobulation, perivascular spaces, and admixed immature T cells. Thymomas are classified into several subtypes according to the morphology of the tumor cells and the proportion of associated immature T cells (i.e., type A, AB, B1, B2, B3, and other rare subtypes) (1).

One of the unique features of thymomas is their frequent association with autoimmune diseases, especially myasthenia gravis (MG). As comprehensively described in other articles in this issue, MG is characterized by autoantibodies against components of the neuromuscular junction and divided into several subgroups based on clinical features and the causative antibody. The subgroup that is associated with thymoma and almost consistently with anti-acetylcholine receptor (AChR) antibodies, is termed thymoma-associated MG (TAMG) (2).

Taking into account that most thymoma subtypes contain immature T cells and are likely involved in the “education” of such T cells like the normal thymus, it has been hypothesized that the “non-tolerogenic” microenvironment in thymomas plays a key role in the pathogenesis of TAMG. Indeed, MG+ thymomas of all major histotypes (except for type A thymomas) contain significantly more mature “pre-emigrant” CD4+ T cells than MG- thymomas (3). Besides, the polymorphism of the non-MHC gene, *CTLA4* that affects T cell receptor signaling appears to correlate with TAMG (4). On the other hand and again across all major histotypes, almost all thymomas show a reduced intratumoral generation of regulatory T cells (Tregs) (5), attenuated MHC class II expression (6), and deficient expression of the autoimmune regulator, AIRE irrespective of MG status (7). Together, these findings suggest that CD4+ effector T cells that mature inside the abnormal microenvironment of thymomas and egress from them to the blood are critical to the development TAMG in thymopoietically active thymomas. Also, a recent comprehensive analysis of thymic epithelial tumors conducted as TCGA (The Cancer Genome Atlas) project has reported meaningful findings associated with TAMG, such as the higher prevalence of aneuploidy and overexpression of genes with sequence similarity with *CHRNA1, TTN*, and *RYR1/RYR2* (8), all of which code for skeletal muscle antigens that are key autoantibody targets in TAMG, i.e., the α-subunit of the AChR, titin and ryanodine receptors, respectively (2). In spite of this progress, the underlying mechanisms leading to the above mentioned common features of MG-associated thymomas have remained largely enigmatic. Moreover, despite the molecular and morphological diversity among thymoma histotypes (8), the hypothesis has not been thoroughly addressed that the underlying mechanisms leading to TAMG might have histotype-specific facets. To test this, we re-analyzed the aforementioned TCGA data sets of thymomas (8) after stratification for thymoma histotype. Since the TCGA study did not reveal TAMG-associated “immune signatures” across the whole thymoma cohort (8), we here focused on histotype-specific enrichments of immunologically relevant pathways in association to TAMG.

## Materials and Methods

### Access to the TCGA Thymoma Data Set

We analyzed the TCGA data set, “Thymoma, PanCancer Atlas,” through the CBioPortal database (http://www.cbioportal.org/), following the final diagnoses submitted by Radovich et al. (8).

### Selection and Stratification of Thymomas in the TCGA Thymoma Data Set

To simplify the analysis of this highly heterogeneous thymoma cohort (8), we focused our stratification on the two most prevalent thymoma subtypes, type AB (*N* = 47) and B2 (*N* = 25) thymomas. This choice was also motivated by the fact that among the thymoma subtypes that are often accompanied by MG [i.e., AB, B1, B2, and B3 thymomas (1)], the differences between AB and B2 thymomas in terms of epithelial morphology, genotype, and gene expression signatures are highly significant, while the abundance of intratumorous, non-neoplastic immature T cells *on average* is comparable (8).

Still, the content of non-neoplastic, immature T cells can be quite variable among type AB thymomas as well as B2 thymomas and this variability may potentially obscure differences between the neoplastic epithelial cells of MG+ and MG- thymomas. Therefore, we divided each of the cohorts of type AB and B2 thymomas further into an immature T lymphocytes-high and immature T lymphocyte-low subgroup based on the mRNA expression levels of TdT (terminal deoxynucleotidyl transferase), i.e., a *bona fide* marker gene of immature T lymphocytes in the thymus. To this end, we first calculated the mean of the normalized counts (~9,743) from all thymoma samples and then chose 10,000 as the cutoff for low (<10k) and high (>10k) subgroups. Among type AB thymomas, this strategy resulted in a TdT-high subgroup that contained 4 MG+ and 21 MG- cases, and a TdT-low subgroup that contained 4 MG+ and 18 MG- cases. In type B2 thymomas, the TdT-high subgroup contained 8 MG+ and 4 MG- cases, and the TdT-low subgroup contained 6 MG+ and 7 MG- cases (Figure S1).

### Differential Expression Analysis

An ANOVA was performed to identify differential expressed genes using a commercial software package SAS JMP11 Genomics, version 7, from SAS (SAS Institute, Cary, NC, USA). A false positive rate of a = 0.05 with FDR correction was taken as the level of significance. For the comparison of the gene expression levels of NEFL, NEFM, CHRNA1, RYR3, SLCO1A2, and PRAME between MG+ and MG- groups (Figure S2), we used the Wilcoxon test with JMP14 (SAS, Cary, North Carolina, USA). Differences at *P* < 0.05 were considered to be significant.

### Analysis of Functional Pathways

Based on the differentially expressed genes between the various MG+ and MG- subgroups of the cohorts of type AB and B2 thymomas, significantly upregulated or downregulated pathways in each MG+ subgroup were extracted, using the KEGG database (https://www.genome.jp/kegg/kegg_ja.html). Because of the particular immunological perspective of our analysis, the differentially expressed genes were also mapped on the extensive collection of inflammation-related pathways that was described by Shen and coworkers (9) after the publication of the TCGA thymoma paper (8). Then, Gene Set Enrichment Analysis (GSEA) was used to determine whether defined lists (or sets) of genes exhibit a statistically significant bias in their distribution within a ranked gene list using the *R* software-packages EnrichmentBrowser (10). The genes were ranked due to their *t*-value based on the comparison between MG+ vs. MG- subgroup. The study was performed under the approval of the Medical Ethics Committee II, Medical Faculty Mannheim, Heidelberg University.

## Results

### Differentially Upregulated Functional Pathways in MG+ Compared to MG- Thymomas

**Figure 1:**
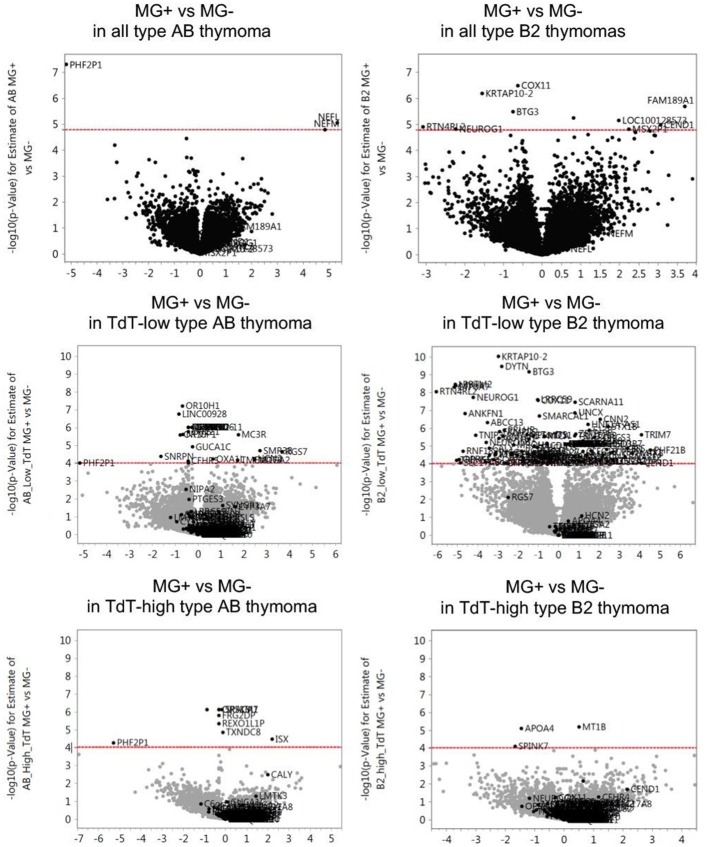
Differentially expressed genes between MG+ and MG- thymomas in type AB and B2 thymomas. For each gene the -log10 (*p*-value) for the difference of gene expression levels between MG+ and MG- groups (vertical axis) is plotted against its log2(fold change) relative expression level of the MG+ group compared to the MG- group (horizontal axis). The dashed red line represents the statistical significance threshold (*P* ≤ 0.05 after adjustment with False Discovery Rate). In both type AB and B2 thymomas, the number of genes that were differentially expressed between MG+ and MG- cases was higher in each of the TdT (terminal deoxynucleotidyl transferase, a *bona fide* marker gene of immature T lymphocytes)-low and TdT-high subgroups than in the non-stratified, i.e., total cohorts of AB and B2 thymomas. The differences between MG+ and MG- cases were particularly obvious in both TdT-low subsets.

According to the stratification of the thymoma cohorts described in Materials and Methods, the following MG+ and MG- thymoma subgroups were compared in terms of gene expression followed by the extraction of functional pathways: (1) all type AB thymomas, (2) all type B2 thymomas, (3) TdT-low type AB thymomas, (4) TdT-high type AB thymomas, (5) TdT-low type B2 thymomas, and (6) TdT-high type B2 thymomas. In both type AB and B2 thymomas, the number of genes that were differentially expressed between MG+ and MG- cases was higher in each of the TdT-low and TdT-high subgroups (subgroups 3–6) than in the non-stratified, i.e., total cohorts of AB and B2 thymomas (subgroups 1 and 2). The differences between MG+ and MG- cases were particularly obvious in both TdT-low subsets (Figure 1). The pathways that were significantly upregulated or downregulated (in the same manner) in both TdT-low and TdT-high subgroups are shown in Table 1. Then, we evaluated whether these pathways were also shared between the AB and B2 thymoma cohorts as shown next.

**Table 1:** Upregulated or downregulated KEGG pathways in myasthenia gravis (MG).

**Name**	**Main_Category**	**Sub_Category**	**NES**	**P-value**
**Upregulated pathways in MG(+) cases in type AB thymoma**				
***hsa00190_Oxidative_phosphorylation***	1. Metabolism	1.2. Energy metabolism	2,57	0,0048
***hsa05012_Parkinson_disease***	6. Human Diseases	6.4. Neurodegenerative diseases	2,45	0,0048
***hsa05010_Alzheimer_disease***	6. Human Diseases	6.4. Neurodegenerative diseases	1,985	0,0048
hsa05016_Huntington_disease	6. Human Diseases	6.4. Neurodegenerative diseases	1,985	0,0048
hsa04932_Non-alcoholic_fatty_liver_disease_(NAFLD)	6. Human Diseases	6.7. Endocrine and metabolic diseases	1,875	0,0048
hsa04714_Thermogenesis	5. Organismal Systems	5.10. Environmental adaptation	2,02	0,0050
hsa03050_Proteasome	2. Genetic Information Processing	2.3. Folding, sorting and degradation	2,03	0,0124
hsa04723_Retrograde_endocannabinoid_signaling	5. Organismal Systems	5.6. Nervous system	1,51	0,0221
hsa03010_Ribosome	2. Genetic Information Processing	2.2. Translation	2,47	0,0247
hsa04260_Cardiac_muscle_contraction	5. Organismal Systems	5.3. Circulatory system	1,7	0,0267
**Downregulated pathways in MG(+) cases in type AB thymoma**				
hsa04520_Adherens_junction	4. Cellular Processes	4.3. Cellular community - eukaryotes	−1,905	0,0048
hsa04933_AGE-RAGE_signaling_pathway_in_diabetic_complications	6. Human Diseases	6.7. Endocrine and metabolic diseases	−1,84	0,0061
hsa04350_TGF-beta_signaling_pathway	3. Environmental Information Processing	3.2. Signal transduction	−1,745	0,0065
hsa05205_Proteoglycans_in_cancer	6. Human Diseases	6.1. Cancers: Overview	−1,65	0,0149
hsa05140_Leishmaniasis	6. Human Diseases	6.10. Infectious diseases: Parasitic	−1,695	0,0178
hsa05206_MicroRNAs_in_cancer	6. Human Diseases	6.1. Cancers: Overview	−1,58	0,0226
hsa05145_Toxoplasmosis	6. Human Diseases	6.10. Infectious diseases: Parasitic	−1,66	0,0248
hsa04062_Chemokine_signaling_pathway	5. Organismal Systems	5.1. Immune system	−1,485	0,0251
hsa05200_Pathways_in_cancer	6. Human Diseases	6.1. Cancers: Overview	−1,445	0,0254
**Upregulated pathways in MG(+) cases in type B2 thymoma**				
hsa04740_Olfactory_transduction	5. Organismal Systems	5.7. Sensory system	1,92	0,0130
**Downregulated pathways in MG(+) cases in type B2 thymoma**				
hsa04141_Protein_processing_in_endoplasmic_reticulum	2. Genetic Information Processing	2.3. Folding, sorting and degradation	−1,88	0,0119
hsa01100_Metabolic_pathways	1. Metabolism	1.0 Global and overview maps	−1,36	0,0126
hsa03030_DNA_replication	2. Genetic Information Processing	2.4. Replication and repair	−2,07	0,0137
***hsa00190_Oxidative_phosphorylation***	1. Metabolism	1.2. Energy metabolism	−1,725	0,0148
***hsa05012_Parkinson_disease***	6. Human Diseases	6.4. Neurodegenerative diseases	−1,66	0,0204
***hsa05016_Huntington_disease***	6. Human Diseases	6.4. Neurodegenerative diseases	−1,56	0,0218
hsa00240_Pyrimidine_metabolism	1. Metabolism	1.4. Nucleotide metabolism	−1,645	0,0246

*NES, normalized enrichment score; P-value, adjusted P-value; Both NES and P-value are mean score (or value) between that of TdT-low and -high groups*.

### Type AB and B2 Thymomas Upregulate Different Functional KEGG Pathways in Relation to their MG Status

When focusing on functional pathways derived from the KEGG database (*N* = 310), 19 functional pathways (6%) showed an MG association in AB thymomas: ten pathways, such as those related to Oxidative phosphorylation, Parkinson disease, and Alzheimer disease, were significantly upregulated, while nine pathways, such as those related to Adherens junction, AGE-RAGE signaling, and TGF-beta signaling, were significantly downregulated in MG+ compared to MG- type AB thymomas. In type B2 thymoma, only eight functional pathways showed an MG association: the pathway related to Olfactory transduction was significantly upregulated in MG+ cases, while seven pathways, such as Protein processing, Metabolism, and DNA replication, were downregulated in MG+ B2 thymomas (Table 1). The respective upregulated and downregulated pathways were not overlapping between type AB and B2 thymomas.

### Type AB and B2 Thymomas Are Differentially Enriched in Inflammation Related Pathways

Taking into account that inflammatory features of the thymoma microenvironment could be involve in the pathogenesis of TAMG, we next focused on pathways (*N* = 70) that are related to inflammation, including features of tumor-infiltrating immune cells and cytokines (9). We identified three pathways, TNF-alpha signaling, MacTh1 cluster, and Chemokine signaling that were significantly downregulated in MG+ type AB thymoma. No pathways were significantly upregulated in MG+ type AB thymoma. In contrast, five pathways, such as those related to T cell cluster, MacTh1 cluster, and LCK median, were significantly upregulated in MG+ type B2 thymoma (Table 2).

**Table 2:** Upregulated or downregulated “immune” pathways in myasthenia gravis (MG).

**Name**	**NES**	***P*-value**
**Suppressed pathways in MG(+) cases in type AB thymoma**		
HALLMARK_TNFA_SIGNALING_VIA_NFKB	−2.09	0.0015
***MacTh1_cluster***	−2.12	0.0020
KEGG_CHEMOKINE_SIGNALING_PATHWAY	−1.535	0.0070
**Activated pathways in MG(+) cases in type B2 thymoma**		
T_Cell_cluster_Iglesia	2.315	0.0031
***MacTh1_cluster***	2.24	0.0031
LCK_Median	2.07	0.0031
UNC_MCD3_CD8	2.015	0.0050
CD8_cluster	2.145	0.0054

*NES, normalized enrichment score; P-value, adjustd P-value*.

*Both NES and P-value are mean score (or value) between that of TdT-low and -high groups*.

### Opposite Enrichment Status of Identical Functional Pathways in MG+ Type AB and B2 Thymomas

**Figure 2:**
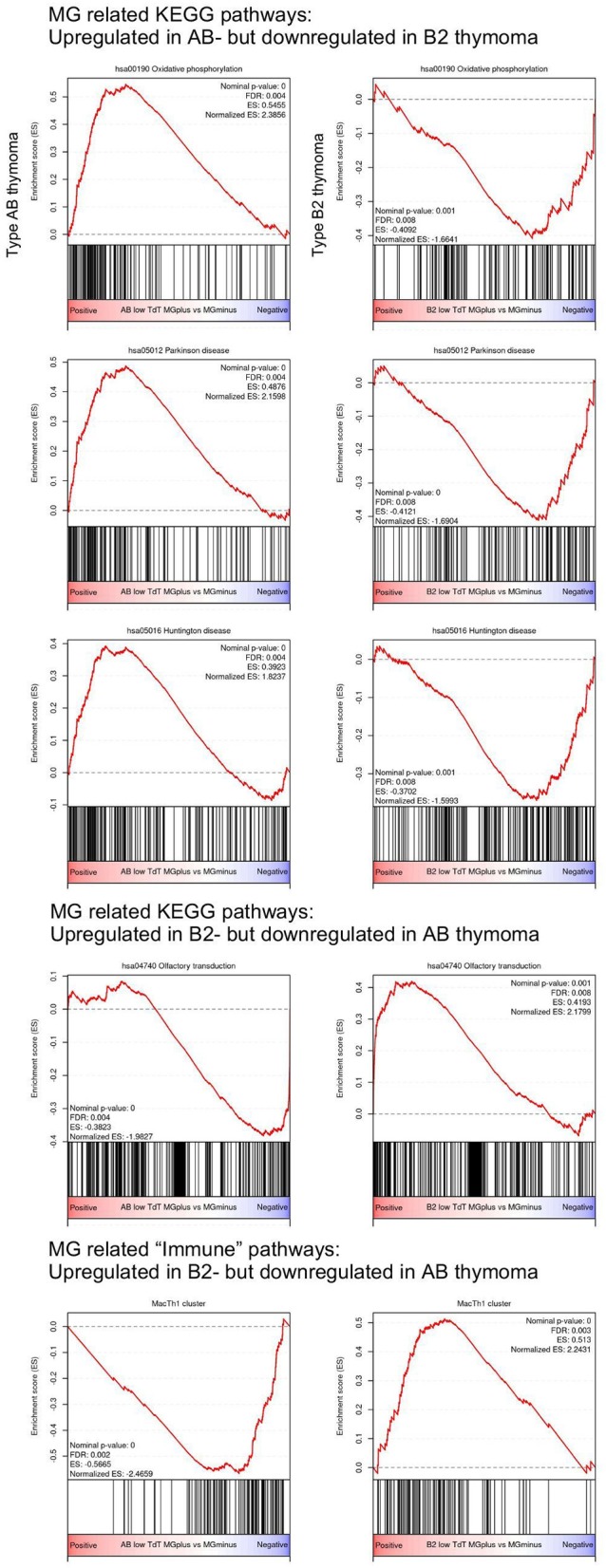
Opposite upregulation/downregulation of MG-associated pathways in type AB and B2 thymomas based on Gene Set Enrichment Analysis (GSEA). Among the KEGG pathway, pathways related to “Oxidative phosphorylation,” “Parkinson disease,” and “Huntington disease” are significantly upregulated in MG+ type AB thymoma, but downregulated in MG+ B2 thymoma. On the other hand, the pathway related to “Olfactory transduction” is significantly downregulated in MG+ type AB thymoma, but upregulated in MG+ B2 thymoma. Among the “immune pathways” provided by Shen et al. (9), the “MacTh1 cluster” is significantly downregulated in type AB thymoma, but upregulated in type B2 thymoma.

Surprisingly, some of the functional pathways that were significantly upregulated in MG+ type AB thymoma were significantly downregulated in MG+ type B2 thymoma: Among the pathways derived from the KEGG dataset, this pattern concerned the pathways of oxidative phosphorylation, Parkinson disease, and Huntington disease (Figure 2 and Table 1). Vice versa, the Olfactory transduction pathway that was significantly upregulated in MG+ type B2 thymomas, was downregulated in MG+ type AB thymomas, although the difference was significant only in the TdT-low subset (and with a minor trend in the TdT-high subset) (Figure 2 and Table S1). Among the “immune pathways,” the “MacTH1 cluster” also followed this pattern (Figure 2, Table 2).

## Discussion

When addressing the question, why some thymomas are accompanied by MG, while others are not, most previous studies have not considered possible pathogenetic differences between the various thymoma histological subtypes (3, 11)—with rare exceptions (12, 13). Here, we have addressed this question separately in type AB and B2 thymomas using the comprehensive and highly reliable TCGA data sets (8). We found that upregulated or downregulated pathways associated with MG were not only barely overlapping between the two subtypes, but for some pathways showed an oppositely enrichment status, i.e., MG-associated pathways that were upregulated in one histotype where downregulated in the other, and vice versa. These observations were more evident in the subgroups of type AB and B2 thymomas, which were poor in non-neoplastic immature, TdT+ T cells. Together, these findings suggest that the mechanisms underlying TAMG might be different in the various thymoma histotypes and mainly operative in neoplastic epithelial cells and/or non-neoplastic mature stromal cells, but not the quantitatively often overwhelming population of immature T cells.

The current study that is based on RNA expression profiles, does not allow to clarify, how the various differentially expressed genes and functional pathways identified here might contribute to TAMG in the two major thymoma histotypes, type AB and B2 thymomas. To truly understand and eventually prove the relevance of the identified pathways for the pathomechanisms leading to TAMG, detailed in situ analyses, the investigation of isolated cell types sorted from fresh thymoma resection specimens and functional studies using *in vitro* or *in vivo* model systems would be necessary. Nevertheless, in face of the fact that TAMG is a neuromuscular disease, it is interesting that some of the identified TAMG-associated KEGG pathways are related to neurodegenerative diseases (Parkinson disease, Alzheimer disease, and Huntington disease). In line with this finding, it has been known for long that expression of neurofilaments in thymomas is associated with TAMG (8, 14), and we show here that this association is strongest in type AB thymomas (Figure S2). Furthermore, expression of the brain-type ryanodine receptor, *RYR3*, in thymomas has been shown to be associated with TAMG (8), but in this case we now find that the association is strongest in type B2 thymomas (Figure S2). Of note, pathways that play a role in the above mentioned neurodegenerative diseases, have been found enriched in a variety of immunobiological settings, including chronic infections, graft-vs.-host disease, cancer biology, cell death, and inflammation (15–17). Likewise, the “Adherens junction” pathway that was the most significantly downregulated TAMG-associated pathway in type AB thymomas (Table 1) has been linked to thymic hypoplasia and lymphopenia (18) and to various autoimmune diseases in conjunction with the leakiness of several blood-tissue and inter-epithelial barriers (19, 20). Considering the quite specific pathology of tumor vessels in the different thymoma subtypes (21), in depth analysis of the tumor vasculature in relation TAMG appears warranted.

Other pathways that have not been linked previously to TAMG to the best of our knowledge are related to metabolism: Oxdative phosphorylation, Protein processing, Metabolic pathways, DNA replication, and Pyrimidine metabolism. Although the mechanisms that link these pathways to TAMG remain enigmatic, it is noteworthy that “Metabolic pathways” and the above mentioned “Alzheimer pathway” are significantly enriched KEGG pathways in Lupus nephritis (22), i.e., in an autoimmmue disease that is often associated with thymomas, though not as commonly as TAMG (23). Similarly, it is unclear how the upregulated “Olfactory transduction” pathway might be linked to the pathogenesis of TAMG in B2 thymomas, but it is interesting that this pathway has been found to be associated with rheumatoid arthritis (24), i.e., another autoimmune disease that occurs in thymoma patients (23).

Completely unexpected was the new finding that among the few identified MG associated pathways (<10% of more than 350 investigated pathways) there was a small subset of 4 pathways that were shared by AB and B2 thymomas, but with diametrically opposed enrichment status in the two tumor types. The detection of these shared but “counter-enriched” pathways and the lack of shared MG-associated “concordantly enriched” pathways among the two thymoma subtypes, lend support to the rational of our “stratification strategy,” to increase the sensitivity of our search for MG-associated pathways by focusing on histologically homogeneous thymoma subtypes. Apart from the above pathways related to neurodegeneration (Parkinson and Huntington disease) and metabolism (Oxidative phosphorylation) (bold/italics in Table 1), one of the eight identified MG-associated inflammatory pathways (9) showed the “counter-enrichment” pattern as well: The MacTh1 cluster-associated gene set was significantly upregulated in TAMG-associated B2 thymomas and downregulated in AB thymomas (bold/italics in Table 2). Imbalanced macrophage polarization is well-known to play an important role in T cell- and autoantibody-mediated autoimmune and allergic diseases (25–28), and can affect T cell apoptosis (29), i.e., a key feature of normal thymic tolerance induction and abnormal thymopoiesis inside TAMG-associated thymomas (30). Furthermore, analyses of MHC class II expression levels and the step-wise maturation of thymocytes inside different thymoma subtypes already gave strong hints that the mechanisms shaping the autoimmune CD4+ T cell repertoire are different in AB and B2 thymomas (31). Accordingly, it is not a priori unreasonable to hypothesize that identical pathways but with opposite enrichment status (e.g., differentially polarized macrophages) could contribute to the same, TAMG-prone phenotype in histologically different thymomas, namely generation of autoreactive CD4+ T cells in very different thymoma microenvironments. Therefore, we deem the MacTh1 cluster-associated gene set a highly promising and potentially informative candidate pathway that warrants in depth comparative analysis in AB and B2 thymomas to elucidate pathogenetic mechanisms leading to TAMG.

In summary, we have identified functional pathways with a significant association with TAMG and, thus, a potential role in its pathogenesis. The identified pathways appear to be mainly operative in cells other than the numerous, thymoma-associated immature T cells, are virtually non-overlapping between type AB and B2 thymomas, and the few shared pathways show diametrically opposite enrichments. These findings parallel the diverse morphology, genetics and global gene expression profiles of AB and B2 thymomas (8, 32). Most previously identified, TAMG-associated genes coded for highly TAMG-specific proteins, such as the acetylcholine receptor itself, or proteins sharing epitopes with TAMG-associated autoantibody targets, such as titin and the ryanodine receptors (8). By contrast, the TAMG-associated pathways detected here appear mostly non-specific, since they are apparently relevant in a variety of other autoimmune diseases (see above). Nevertheless, these pathways appear as promising candidates for future analysis to fill the wide gap of knowledge between the largely enigmatic microenvironments of the various thymoma histotypes and the stereotypic, TAMG-eliciting egress of autoreactive T cells from thymopoietically active, histologically diverse thymomas (31).

## Data Availability Statement

The datasets generated for this study are available on request to the corresponding author.

## Ethics Statement

The study was performed under the approval of the Medical Ethics Committee II, Medical Faculty Mannheim, Heidelberg University (approval # 2015-541N-MA).

## Author Contributions

YY and AM designed the study and wrote the manuscript. CS performed the bioinformatic and statistical analyses. YY, CS, and AM analyzed the data. C-AW, JT, BS, and PS helped with the interpretation of the data under a tumor-biological and neurological perspective. All the authors contributed to the editing of the manuscript, and read and approved the final version before submission.

### Conflict of Interest

The authors declare that the research was conducted in the absence of any commercial or financial relationships that could be construed as a potential conflict of interest.
