# Supplementary material for: Thymoma Associated Myasthenia Gravis (TAMG): Differential Expression of Functional Pathways in Relation to MG Status in Different Thymoma Histotypes
Source: Front Immunol. 2020 Apr 16;11:664. doi: 10.3389/fimmu.2020.00664 (PMC7176899; doi:10.3389/fimmu.2020.00664)
Supplement: Supplementary file 1 [file Data_Sheet_1.PDF]

# *Supplementary Material*

## Supplementary Figures

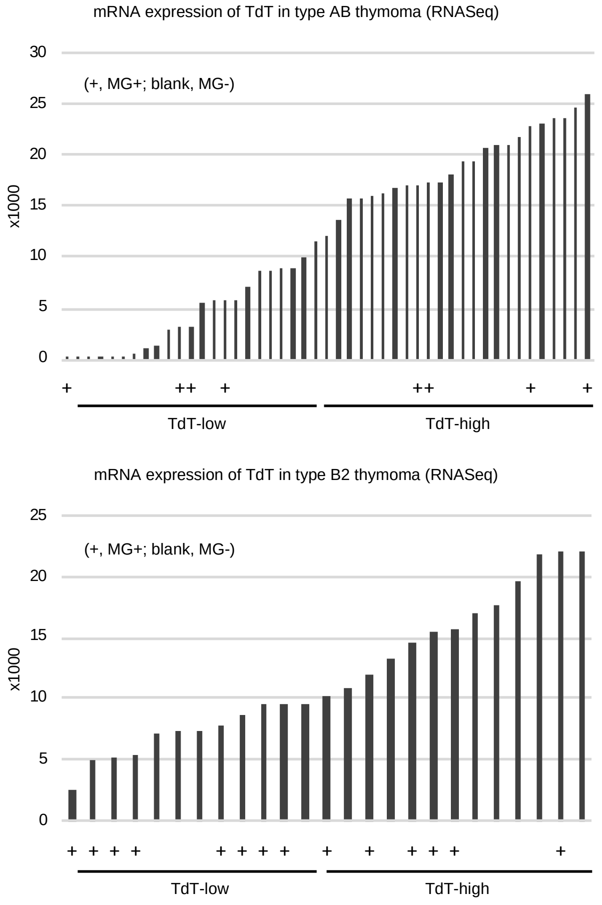


**Supplementary Figure 1 (Figure S1):** Histogram of TdT (terminal deoxynucleotidyl transferase) mRNA expression in type AB and type B2 thymomas. The vertical axis denotes the expression level of TdT (the normalized counts from all thymoma samples). The horizontal axis indicates each case: + means MG+ case, and blank means MG- case.

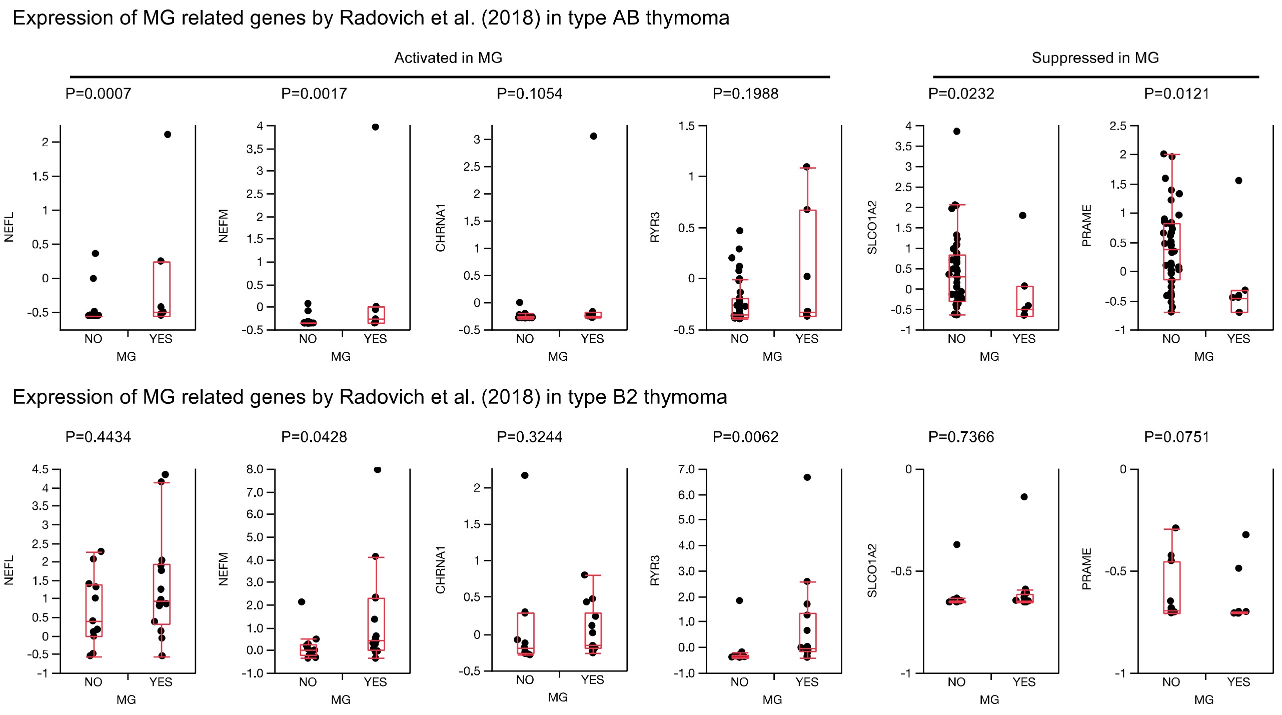


**Supplementary Figure 2 (Figure S2):** Expression of MG related genes as reported by Radovich et al (8) (i.e., NEFL, NEFM, CHRNA1, RYR3, SLCO1A2, and PRAME) after stratification in type AB and type B2 thymomas. The MG association of neurofilaments (NEFL, NEFM) is stronger in type AB than B2 thymomas, and expression of the brain-type ryanodine receptor, RYR3 is stronger in type B2 than AB thymomas.

# Supplementary Table 1 (Table S1)

**Table S1. Downregulation of Olfactory transduction pathway in MG+ type AB thymoma**

| Name | Main_Category | Sub_Category | Group | NES | adjusted_pvalue |
|---|---|---|---|---|---|
| hsa04740_Olfactory_transduction | 5. Organismal Systems | 5.7. Sensory system | Type AB, TdT-low | -1.98 | 0.0040 |
| hsa04740_Olfactory_transduction | 5. Organismal Systems | 5.7. Sensory system | Type AB, TdT-high | -1.16 | 0.2831 |

NES, normalized enrichment scores
